# Observations on the Ulcerative Process, and Its Treatment, Particularly When Affecting the Leg

**Published:** 1834-04-01

**Authors:** 


*Observations on the Ulcerative Process, and its Treatment, particularly when affecting the Leg.* By Wm. Eccles, Surgeon.— London, 1834. 8vo. pp. 66.

This essay is so exceedingly short, that it must be considered rather as a specimen of Mr. Eccles' style, than as a practical treatise on ulcers. We do not wish to discourage his attempts,—far from it; nor do we intend to pen a panegyric on prolixity; but there is a certain point below which a subject cannot be abridged, without forming notes for a book, rather than a real work. The following extract (which occupies five pages of the original,) will show, however, that

Mr. Eccles writes vigorously and pleasantly enough, as far as he goes.

"Surgeons, in general, are ignorant of the manner in which bandaging operates so beneficially. Nor do we find that authors throw much light on the matter. Even John Bell says no more than that it acts by supporting the veins; but he does not show how this support causes flesh to grow, nor even how it operates to relieve that distended condition of the vessels which is assumed to exist in the diseases under consideration.

"A modern author affirms, after stating that chronic inflammation 'consists in a dilated and feeble state of the venous circulation, accompanied by increased arterial action, the result of which is that the blood-vessels are unable to propel their contents,' that bandaging, acting as a mechanical support, 'restores to the vessels the power of propelling the fluid along their canals.'

"Mr. Baynton tells us that, in the inflammation attending ulcers, 'the parts are supplied with a larger quantity of blood than was furnished in a state of health; this, under the peculiar circumstances of the arteries, will occasion a greater deposition of lymph between the interstices of the muscles and in the cells of the cellular membrane than is necessary for their lubrication, or than the absorbents can carry away, which, gradually increasing, will remove the absorbents from their vicinity to the arteries, and consequently occasion a loss to them of the effect of arterial impulse which, while the vascular system of the limb continues in its perfect state, may be supposed to have considerable effect in propelling the returning lymph, as the lymphatic vessels are plentifully supplied with valves. Therefore, I conclude that the principal difficulty which occurred in the curing of ulcers has been occasioned by deficiency of power in the absorbent vessels.'

"If we throw aside all speculation which is based on such assumption as, that the arteries act more or less in inflammation, that the veins propel the blood, or merely convey it, that absorption is promoted by arterial action, the following appears to be the plain state of the case. The skin is more distended with blood than is natural, and bandaging tends to squeeze this fluid from its vessels, and consequently to diminish the cutaneous circulation. This explanation rests for proof on the evidence of our senses: the inflamed limb is distended, since by actual admeasurement we find it enlarged; it is distended by blood, because we find no other source for an accretion of matter except that fluid or the serum derived from it; it moreover demonstrates its presence to us by the redness of the skin, and by its unusual flow if this be cut. Lastly, that pressure drives the blood from the cutaneous vessels (be they named arteries, veins, or capillaries,) is evident, since, when we press our hands upon the skin, this becomes pale, and when we remove them it again reddens.

"We may say, positively, then, that a bandage presses the blood out of the cutaneous vessels. It will be naturally asked, where does that fluid rush to? do you impede the circulation? The answer is, no; there is no impediment to the flow of blood through the limb generally, because the deep-seated arteries and veins carry that on with their usual facility; nor can there be any hindrance to the passage of blood from the larger cutaneous branches, because they have numerous anastomoses with those more deeply seated.

"In this way, then, an ulcer is relieved of the chronic inflammation: viz. by the diversion of the blood into another channel. It has been said, that this plan therefore should cure active inflammation; but this does not happen, because experiment shows that, while parts retain their natural vigour, the mechanical effect of pressure in diminishing the quantity of blood is counteracted by a tendency in the vital parts to act with greater force; so that we always find pressure excite inflammation in a healthy part, and increase that action in an organ already actively inflamed. Hence the absurdity of that indiscriminate use of bandages recommended at different periods by Whately, Baynton, and others." (P. 19.)

